# Cutaneous Neuroendocrine Metastases of Visceral Origin Responsive to Surgical Resection and Targeted Radionuclide Therapy

**DOI:** 10.1155/2024/8873822

**Published:** 2024-02-06

**Authors:** Eleanor Tung-Hahn, Ghassan El-Haddad, Jonathan Strosberg

**Affiliations:** ^1^Lake Erie College of Osteopathic Medicine (LECOM), 5000 Lakewood Ranch Blvd, Bradenton, FL 34211, USA; ^2^Moffitt Cancer Center, Department of Diagnostic Imaging and Interventional Radiology, 12902 USF Magnolia Drive, Tampa, FL 33612, USA; ^3^Moffitt Cancer Center, Department of Medical Oncology and Hematology, 12902 USF Magnolia Drive, Tampa, FL 33612, USA; ^4^University of South Florida College of Medicine, Department of Oncologic Sciences, 560 Channelside Dr, Tampa, FL 33602, USA

## Abstract

Neuroendocrine neoplasms (NENs) encompass a diverse range of biologically and behaviorally distinct epithelial malignancies that derive from neuroendocrine cells. These neoplasms are able to secrete a variety of bioactive amines or peptide hormones. The majority of NENs are well-differentiated and are defined as neuroendocrine tumors (NETs). While NETs are known to frequently metastasize to lymph nodes, liver, and lungs, spread to the skin is extremely rare and is often a late finding. Because cutaneous metastasis from a visceral site represents distant tumor dissemination, prompt histologic diagnosis is critical in terms of selecting further treatment options and ultimately impacts subsequent prognosis. This report presents a man with painful cutaneous NET metastases initially on the face then scalp. He had a prior history of longstanding and progressive stage IV visceral disease. Multimodal therapy with initial surgical resection of the larger facial lesion and radionuclide infusion therapy was undertaken. Excision fully removed the temple lesion and resolved pain. Peptide receptor radionuclide therapy (PRRT) with ^177^Lu-DOTATATE, a radiolabeled somatostatin analog that targets somatostatin receptors on NETs, was given along with maintenance lanreotide therapy, which resolved the scalp lesion, prevented recurrence of prior lesions and development of new cutaneous metastases, and controlled his visceral disease. PRRT has not been previously described in the management of cutaneous NET metastases. Due to the rare nature of cutaneous NET metastases, there is no consensus regarding optimal management. As such, we propose novel multimodal therapy involving excision and targeted radionuclide therapy as a possible effective option.

## 1. Introduction

Neuroendocrine neoplasms (NENs) are a heterogeneous collection of epithelial malignancies that originate from neuroendocrine cells, mainly within the gastrointestinal and bronchopulmonary systems [1]. These cancers possess the ability to produce bioactive amines and/or peptide hormones and have secretory granules and synaptic-like vesicles [1, 2]. Most NENs are characterized as well-differentiated and are classified as neuroendocrine tumors (NETs). While NETs frequently spread to the liver, lymph nodes, and bone, metastases to the skin are very uncommon and often are a late manifestation of advanced disease [3, 4]. If cutaneous NET metastases do occur, they are typically solitary, subcutaneous papules or nodules [3, 5]. We present a case of two eruptive painful cutaneous NET metastases occurring in a patient with prior stage IV visceral disease successfully managed with resection and targeted ^177^Lu-DOTATATE combined with monthly maintenance lanreotide therapy.

## 2. Case Report

A 71-year-old male (Fitzpatrick skin type 1) with metastatic small bowel NET presented for routine full skin examination with dermatology. He had a prior dermatologic history of basal cell carcinoma, actinic keratoses, and an atypical nevus. He noted new tender lesions on his left temple and nasal tip that had been present for a few months. His self-reported past medical history was significant for hypertension and benign prostatic hyperplasia.

**Figure 1:**
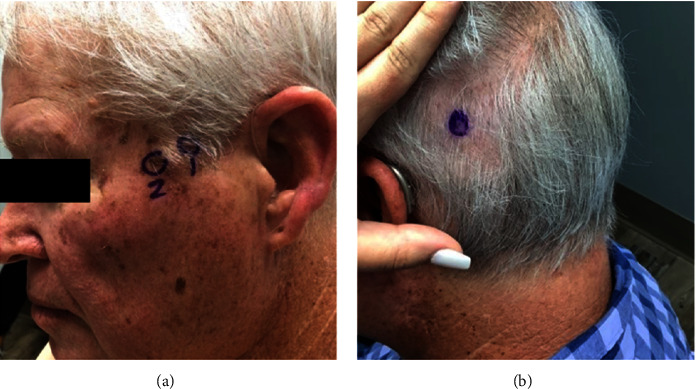
(a) Preoperative left posterior temple labeled #1-small flesh-colored subcutaneous papule-biopsy proven cutaneous NET metastasis (labeled #2-biopsy proven seborrheic keratosis). (b) Prebiopsy left central parietal scalp-small flesh-colored subcutaneous papule -biopsy proven cutaneous NET metastasis with positive peripheral and deep margins.

On physical examination, significant findings included a 3 mm flesh colored subcutaneous papule within his hair on the left posterior temple #1 (Figure 1(a)), a scaly brown verrucal plaque on the left posterior temple #2, and a pearly 3 mm flesh-colored papule on his nasal tip. These lesions were shave biopsied for definitive diagnoses.

**Figure 2:**
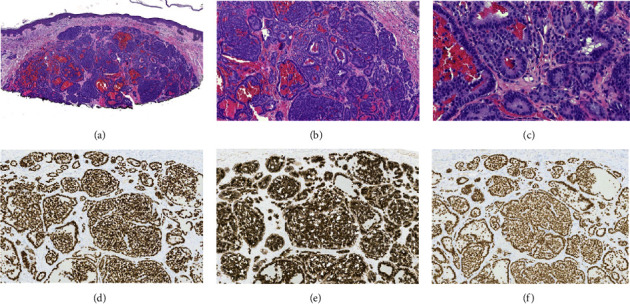
(a–c) Hematoxylin and eosin staining of the left temple biopsy at low (a), medium (b), and high (c) power. (d–f) Immunostaining of the left temple biopsy using INSM-1 (d), CK (e), and CDX2 (f).

Histopathological examination of two specimens revealed common lesions: a nodular basal cell carcinoma (nasal tip) later treated with Mohs micrographic surgery and seborrheic keratosis (left posterior temple #2). Analysis of the left posterior temple lesion #1 revealed a well-circumscribed and focally infiltrative dermal nodule composed of closely packed nests and ductal appearing/pseudorosetting structures. Tumor cells were cuboidal to columnar with ample amount of amphophilic cytoplasm, round nuclei, and powdery chromatin. Nuclear atypia was mild, and mitoses were rare (Figures 2(a)–2(c)). The lesion was diffusely positive for a cytokeratin cocktail (Figure 2(e)), insulinoma-associated protein 1 (INSM-1, Figure 2(d)), and homeobox protein CDX-2 (CDX2, Figure 2(f)). It was diffusely negative for cytokeratin 7 (CK7), cytokeratin 20 (CK20), carcinoembryonic antigen (CEA), special AT-rich binding protein (SATB2), prostatic tumor suppressor gene (NKX3.1), prostate specific membrane antigen (PSMA), thyroid transcription factor-1 (TTF1), GATA binding protein 3 (GATA3), and tumor protein 63 (a nuclear marker of myoepithelial cells (P63)). Albumin in situ hybridization was also negative. Lack of CEA staining demonstrated the absence of true ductal differentiation. Based on these findings, a final diagnosis of metastatic well-differentiated gastrointestinal NET extending to deep margin was rendered.

After discussion of his biopsy results, he elected to proceed with wide local excision for the metastatic NET. During that visit, he identified a new tender 2 mm flesh colored papule on his left central parietal scalp (Figure 1(b)) that had appeared in the intervening 2-week period. The lesion on his left temple was widely excised and found to have residual metastatic well-differentiated NET with clear margins and an incidental apocrine hidrocystoma, intradermal nevus, and seborrheic keratoses. The punch biopsy of the new parietal scalp lesion was also diagnosed as a metastatic well-differentiated NET extending to peripheral and deep margins.

### 2.1. Patient's NET History

This patient presented with symptoms of facial flushing beginning seventeen years earlier. One year after these symptoms began, he was evaluated by his primary care physician for treatment-resistant hypertension and underwent a CT scan incidentally revealing a suspicious mass in his right psoas muscle. Subsequent biopsy at a local hospital was consistent with well-differentiated NET. A follow-up MRI demonstrated a small bowel lesion. He later underwent a right hemicolectomy and excision of the mass in his psoas muscle. Pathology from the terminal ileum revealed well-differentiated NET with 2/11 positive lymph nodes. Pathology from the right psoas resection was also consistent with metastatic NET extending to the margins of excision.

Six years later, he sought consultation and care at a multidisciplinary neuroendocrine oncologic center regarding further management of his disease. Baseline laboratory testing revealed an elevated level of 5-HIAA in a 24-hour urine sample (35.3) and an elevated serum chromogranin level (50). Repeat CT scan of the abdomen and pelvis identified a new 4 cm hypodense lesion in the left hepatic lobe. Octreoscan confirmed a large mass in the left lobe of the liver as well as additional foci in the mesentery and iliopsoas region. Left hepatic lobectomy and wedge resection of sections 4B and 6, cholecystectomy, and radiofrequency ablation of liver metastases × 3 were performed. Pathologic findings were consistent with metastatic high-grade NET with ki-67 of 22%.

**Figure 3:**
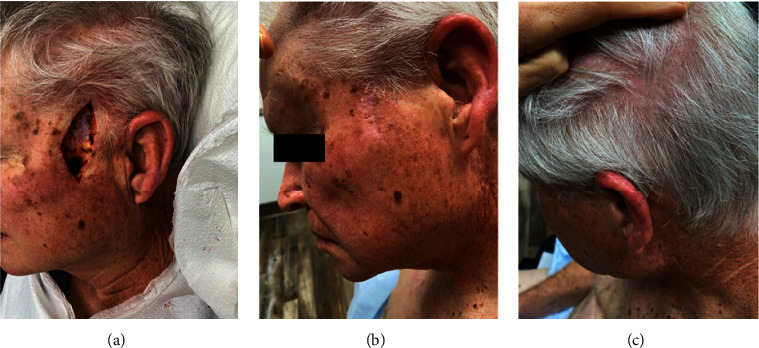
(a) Postexcision of left posterior temple (lesion #1 in Figure 1(a)): residual well-differentiated NET metastasis with clear margins. (b) 6 month follow-up postexcision of the left posterior temple lesion. (c) 6 months postbiopsy left central parietal scalp-lesion (lesion shown in Figure 1(b)) clinically resolved after PRRT.

Five years later, he developed new liver metastases and began treatment with the somatostatin analog (SSA) lanreotide. After only five months on this therapy, he was noted to have hepatic progression. He underwent 5 transarterial chemoembolization (TACE) procedures over 1 year. To address an enlarging periaortic lymph node, he was treated with additional focal radiation. His hepatic metastases progressed further, as seen on ^64^Cu-DOTATATE PET scan, and he was treated with ^177^Lu-DOTATATE, a radiolabeled somatostatin analog. His metastatic skin lesions were diagnosed as he was being scheduled for this treatment. Over the course of 6 months after his excision, he underwent 4 infusions of ^177^Lu-DOTATATE, a radiolabeled SSA representing a form of peptide receptor radionuclide therapy (PRRT). This treatment was well-tolerated. During this therapy, the parietal scalp metastatic lesion resolved and there was no recurrence of the left temple lesion (Figures 3(a) and 3(b)). To date, he continues to receive monthly lanreotide injections as part of his maintenance treatment plan and remains free of recurrence. A repeat PET scan, which was performed during this interval period, demonstrated stable disease.

## 3. Discussion

NENs can vary in their biologic behavior and response to treatment. While metastasis to the lymph nodes, liver, and lung is frequently seen, spread to the skin is exceedingly uncommon [5, 6]. There have only been 45 cases of cutaneous NEN metastases reported in the literature (including this one) [2, 5, 7]. Typically, NET metastases are clinically nondescript and appear as nonulcerated subcutaneous papules or nodules [3, 5]. Clinical suspicion for cutaneous metastasis should be high if any new, changing, or symptomatic lesions of any size are encountered on physical examination in a patient with metastatic NET. Due to the uncommon occurrence of cutaneous spread of NETs, there are no explicit guidelines regarding preferred treatments. Management of metastatic NETs that have spread to the skin should be tailored to the individual based on the patient's overall disease manifestations. In our case, multimodal procedures and therapies were employed to control his visceral, hepatic, and rapidly appearing cutaneous metastases. Overall prognosis differs widely amongst NETs due to the continuum of biologic behavior.

From the literature, one small study commented on the clinical behavior and proposed management of symptomatic cutaneous NET metastases [2]. Zuetenhorst et al. noted that painful NET metastases quickly multiplied, whereas nonpainful lesions remained comparatively indolent. In their cohort, very painful metastases were resistant to medical analgesic therapy, but long-term palliation of pain was achieved with local excision [2].

Somatostatin analogs (SSAs) have long been used as maintenance therapy to suppress the symptoms of carcinoid syndrome (if present as in his case) and inhibit tumor growth [8–10].

Systemic treatment of NETs with ^177^Lu-DOTATATE is approved for patients with progressive disease and improves progression-free survival by 79% when compared to long-acting octreotide, an SSA [11, 12]. PRRT has not been previously described in the management of cutaneous NET metastases. Our case report suggests that ^177^Lu-DOTATATE may be efficacious for treatment of cutaneous NET metastases. While this outcome for management of cutaneous NET metastases is encouraging, a subsequent study with a longer period of observation would be necessary for generalizability and standardization of treatment.

## 4. Conclusion

Cutaneous NET metastases are an uncommon occurrence and are often a late finding in advanced disease. There is not yet a consensus regarding optimal treatment of cutaneous NET metastases. Our case highlights innovative use of sequential, multimodal interventions as an effective possible option. In this patient with two symptomatic, cutaneous NET metastases, surgical resection effectively removed and resolved pain in the larger, tender facial lesion. Subsequent systemic PRRT with ^177^Lu-DOTATATE effectively treated the remaining cutaneous lesion.

## Data Availability

All data supporting the conclusions are included within the article.
